# Verbal Memory Decline following DBS for Parkinson’s Disease: Structural Volumetric MRI Relationships

**DOI:** 10.1371/journal.pone.0160583

**Published:** 2016-08-24

**Authors:** Ruben Geevarghese, Daniel E. Lumsden, Angela Costello, Natasha Hulse, Salma Ayis, Michael Samuel, Keyoumars Ashkan

**Affiliations:** 1 Department of Neurosurgery, King’s College Hospital NHS Foundation Trust, King’s Health Partners, London, United Kingdom; 2 Department of Neurosurgery, Charing Cross Hospital, London, United Kingdom; 3 Complex Motor Disorders Service, Evelina Children’s Hospital, Guy’s & St Thomas’ NHS Foundation Trust, London, United Kingdom; 4 Department of Neuropsychology, King’s College Hospital NHS Foundation Trust, King’s Health Partners, London, United Kingdom; 5 Department of Neurology, King’s College Hospital NHS Foundation Trust, King’s Health Partners, London, United Kingdom; 6 Department of Primary Care and Public Health Sciences, King’s College London, United Kingdom; 7 Clinical Neurosciences, Institute of Psychiatry, Psychology & Neuroscience, London, United Kingdom; University of Toronto, CANADA

## Abstract

**Background:**

Parkinson’s disease is a chronic degenerative movement disorder. The mainstay of treatment is medical. In certain patients Deep Brain Stimulation (DBS) may be offered. However, DBS has been associated with post-operative neuropsychology changes, especially in verbal memory.

**Objectives:**

Firstly, to determine if pre-surgical thalamic and hippocampal volumes were related to verbal memory changes following DBS. Secondly, to determine if clinical factors such as age, duration of symptoms or motor severity (UPDRS Part III score) were related to verbal memory changes.

**Methods:**

A consecutive group of 40 patients undergoing bilateral Subthalamic Nucleus (STN)-DBS for PD were selected. Brain MRI data was acquired, pre-processed and structural volumetric data was extracted using FSL. Verbal memory test scores for pre- and post-STN-DBS surgery were recorded. Linear regression was used to investigate the relationship between score change and structural volumetric data.

**Results:**

A significant relationship was demonstrated between change in List Learning test score and thalamic (left, p = 0.02) and hippocampal (left, p = 0.02 and right p = 0.03) volumes. Duration of symptoms was also associated with List Learning score change (p = 0.02 to 0.03).

**Conclusion:**

Verbal memory score changes appear to have a relationship to pre-surgical MRI structural volumetric data. The findings of this study provide a basis for further research into the use of pre-surgical MRI to counsel PD patients regarding post-surgical verbal memory changes.

## Introduction

Parkinson’s disease (PD) is a chronic, degenerative disorder whose cardinal features include bradykinesia, resting tremor and muscle rigidity [1].

The mainstay of treatment in PD is pharmacological, primarily targeted at increasing dopaminergic activity in the nigro-striatal pathway. However, as the disease progresses, increasing doses are required which are associated with the onset of unwanted drug-induced dyskinesias and motor fluctuations [2].

Deep brain stimulation (DBS) provides a non-pharmacological treatment modality for medically refractory PD patients. DBS involves the insertion of electrodes into a particular sub-cortical area and stimulating it at particular frequencies so as to provide symptomatic relief [3]. The most common targets for DBS in PD are the Subthalamic nucleus (STN) and the globus pallidus interus (GPi). Randomised trials have shown no significant difference in the improvement of motor outcomes comparing DBS of either target [4–5].

DBS has been associated with cognitive changes and these may be attributable to poorly defined patient selection criteria [6], changes in medical therapy, surgical complications [7] or to the stimulation itself. It had been suggested that cognitive changes were more frequent in patients undergoing STN stimulation [4]. However, a recent randomised controlled trial showed no difference in cognitive outcomes between patients undergoing STN or GPi DBS [8].

We have previously reported verbal memory decline in a cohort of patients who underwent bilateral STN DBS [9]. Whilst the reasons for such changes following DBS are complex, correlates between structural brain changes and cognition in PD patients may help predict post-DBS changes in cognitive testing.

Verbal memory impairment has been associated with hippocampal atrophy in an MRI study of PD patients [10]. Whilst not thus far investigated in PD patients, volumetric MRI studies of normal subjects [11] and epileptic patients [12] have demonstrated a relationship between the thalamic volume and verbal memory performance.

Hence, it may be possible that certain pre-disposed individuals are more susceptible to post-surgical verbal memory changes. Thus, the aim of this study was to explore the relationship between pre-surgical thalamic and hippocampal volumes with post-DBS surgery score decline in verbal memory.

## Methods

### Patient selection

A consecutive group of 40 patients with PD was identified retrospectively from the Functional Neurosurgical Movement Disorders database. Patients selected for DBS by the multidisciplinary team were those PD patients with medically refractory disease, including those with motor complications and dyskinesias, compromising their quality of life, without significant neuropsychological or neuropsychiatric impairment, a Levodopa challenge test showing at least 30% improvement and an unremarkable brain MRI scan. To achieve a homogenous patient group, only patients who had undergone Bilateral STN DBS were selected. This was a retrospective study accessing data obtained during routine clinical care of patients. As such based on our institution policy, no ethical approval was required.

Patient demographics including age, gender, duration of diagnosis, UPDRS part III (off) score and handedness was extracted from patient notes. A summary of demographics is included in Table 1. All patients received a post-implantation stereotactic CT to fuse with pre-operative stereotactic MRI verifying the position of active DBS contact in the STN. At follow-up, a satisfactory motor response was observed in all patients in our cohort [13].

**Table 1:** Patient Demographics.

Patient Demographic	
Gender	17 female and 23 male
Handedness	38 right handed and 2 left-handed
Age at surgery (mean ± SD)	60.2 ± 7.2 years old
Age of onset (mean ± SD)	48.4 ± 8.7 years old
Duration of illness (mean ± SD)	11.9 ± 5.0 years
UPDRS Part III Score (mean ± SD) *(n = 38)*	39.2 ± 19.1

Summary of patient demographics. UPDRS Part III score was not available for 2 patients.

### MRI Data acquisition and electrode localisation

Routine preoperative MRI scans were available for all 40 patients. Preoperative stereotactic MRI was performed with a GE (General Electric) 1.5T HD MRI scanner, equipped with Twin Speed gradients. For all patients MRI imaging was performed with Leksell G Frame (Elekta Instrument AB, Stockholm, Sweden). The MRI protocol included a T_1_-weighted 3-dimensional inversion recovery prepared fast spoiled gradient echo volume, with inversion time = 450ms, echo time (TE) = 3.5 ms, repetition time (TR) = 8.4ms, flip angle = 25° and receiver bandwidth (BW) = +/- 23 KHz. The inversion recovery prepared spoiled gradient echo volume images were acquired with field of view = 300 mm, NEX = 1, matrix = 256 x 256, and slice thickness = 1.5 mm. The 3D MR images were transferred offline for subsequent analysis. All patients underwent a post-operative stereotactic CT scan which was fused with the pre-operative stereotactic MRI scan to verify the position of the electrodes. In all cases the electrode position was deemed within the STN target thus not requiring any revisional surgery.

### MRI Image pre-processing

**Fig 1:**
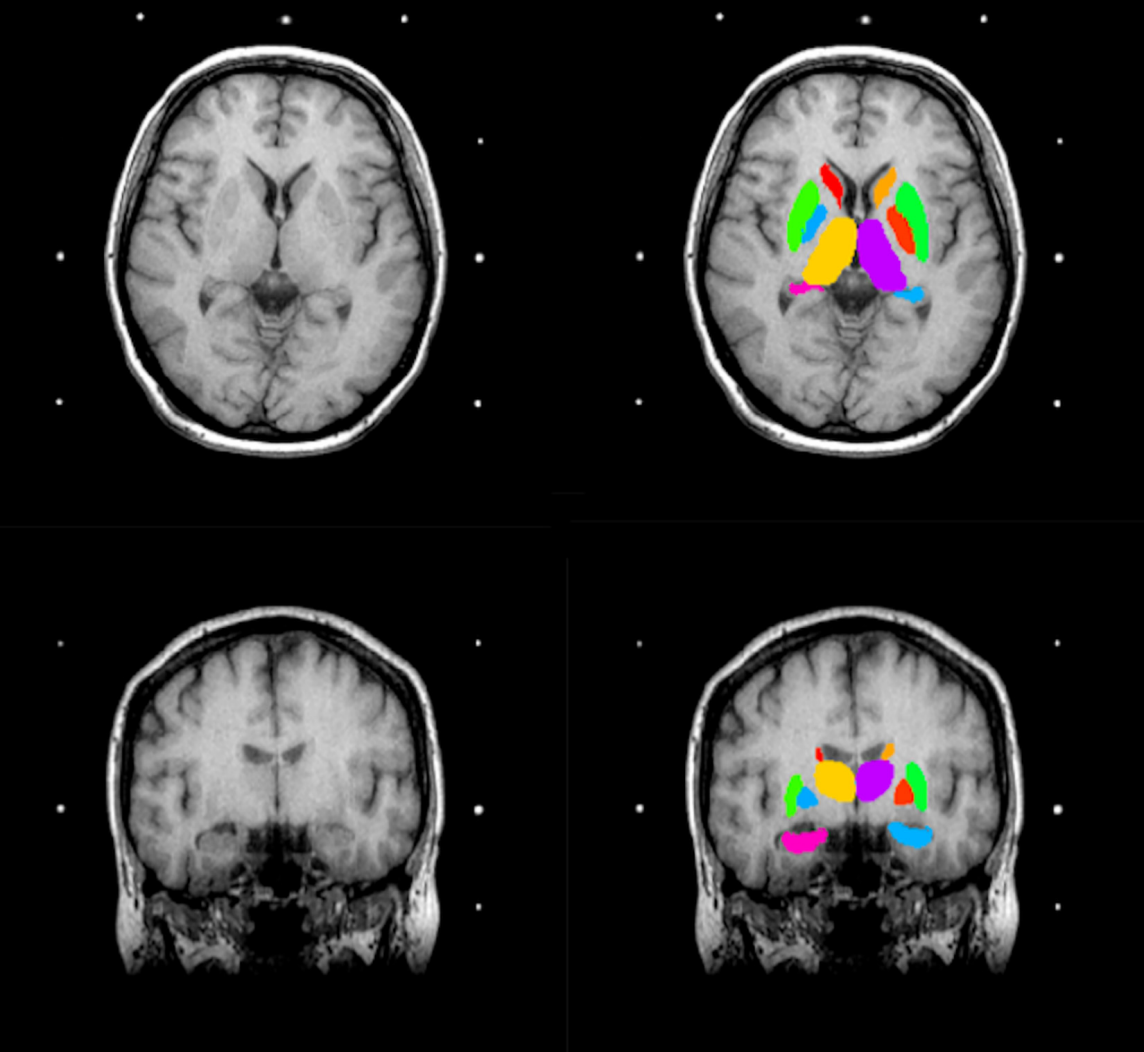
Brain MR images. Axial (top) and coronal (bottom) Brain MR image before (left) and after (right) application of FIRST. The colours correspond to the segmented sub-cortical structures: pink (left hippocampus), light blue (right hippocampus), orange (left thalamus) and purple (right thalamus).

Raw MRI images exported from the MRI scanner were manually assessed to ensure that there was no evidence of major head motion or other image artefacts. All 40 scans passed this assessment. An example of a scan is included in Fig 1.

Skull and neck voxels were removed using the Brain Extraction Tool (BET), part of FMRIB’s Software Library (FSL) (v4.19) [14–15]. Bias field correction was then applied, using FAST, also a part of FSL [16]. The corrected image was then used in subsequent processing steps.

### Structure volume estimation

Two structures were selected, the thalamus and hippocampus. The algorithm FIRST, was applied to separately estimate left and right volumes of each structural region.

FIRST (v1.2) is part of FSL and performs both automated registration and segmentation of the aforementioned structural regions [17]. During registration, the input data (3D T1 images) are transformed to the Montreal Neurological Institute (MNI) 152 standard space, by means of affine transformations based on 12 degrees of freedom (i.e. three translations, three rotations, three scalings and three skews). After structural region registration, a structural region mask is applied, to locate the different structural regions, followed by segmentation based on shape models and voxel intensities. Absolute volumes of the structural region are calculated taking into account the transformations made in the first stage [17]. Finally, a boundary correction is used to determine which boundary voxels belong to the structure or not. In this study a Z-value of 3 was used, corresponding to a 99.998% certainty that the voxel belonged to the structural region.

After registration and segmentation of all 40 MR scans, segmented structural regions were visually checked for errors in registration and segmentation. None were found. An example of structural region segmentation, after boundary correction, is presented in Fig 1. Absolute volumes in millimetres (mm^3^) for left and right thalamus and hippocampus were acquired as calculated by fslstats, a tool part of the FSL Software Library, for each patient.

Total intracranial volume (TIV) was estimated by multiplying the scaling factor for each input scan (generated from SIENAX) and the MNI 152 ICV. SIENAX starts by extracting brain and skull images from the single whole-head input data [15]. The brain image is then affine-registered to MNI152 space [18] (using the skull image to determine the registration scaling); this is primarily in order to obtain the volumetric scaling factor, to be used as a normalisation for head size. Next, tissue-type segmentation with partial volume estimation is carried out [19] in order to calculate total volume of brain tissue (including separate estimates of volumes of gray matter and white matter). The MNI 152 ICV was calculated from a priori standard space maps.

Relative volumes were calculated for each structure by dividing the absolute volume (of the structure) by the TIV (for each patient, obtained from SIENAX) x 100. This gave a volume for each sub-cortical structure expressed as a percentage of TIV. This correction allows for variation in head size between patients. Corrected volumes were used in the subsequent statistical analyses.

### Neuropsychological testing

In this study, verbal memory was assessed pre- and post-DBS surgery [20] using the Birt Memory and Information Processing Battery (BMIPB). All the employed tests have well-validated age-standardised normative data. Neuropsychological tests were administered while the patients were “on” taking their routine medication pre-surgery. The post DBS assessment was carried out in the on-stimulation and on-medication state. Testing was completed on average 6.9 months before (S.D. 2.8 months, Range 3–14 months) and 8.8 months (S.D. 2.0 months, Range 6–16 months) after the surgical procedure.

The BMIPB assessed verbal recall memory through three tests: immediate and delayed story recall and list learning. Parallel versions of the recognition and recall memory tests were given on the post-DBS assessment. Each test in the battery has an age-standardised score with a mean and S.D. of 10 ± 3 respectively. This allowed for comparisons between subjects, independent of age.

### Statistical Analysis

Data was analysed with IBM SPSS Statistics (Version 22.0.0.0). A p value <0.05 was chosen as a criterion to determine significance.

A Wilcoxon signed rank-test was used to assess the difference between pre- and post-DBS scores for each of the three tests, as the normality was not met by the data examined, and hence the paired t-test was considered as inappropriate.

Initially, the association between the change in test score and potential confounders including age, duration of symptoms and disease severity (UPDRS Part III score), was assessed with linear regression modeling. A p value of <0.2 was used for subsequent selection of confounders for the primary analysis.

Linear regression modeling was then used for the primary analysis. The outcome of interest was the absolute change in the test score, and the predictors were the structural volumes of interest and the appropriate significant clinical confounders, as previously determined, for the neuropsychological test of interest.

We also undertook a secondary analysis in which patients were divided into “stable” and “decline” groups for each test. All test scores used for data analysis were based on age-standardised normative data. Hence the standardised score for an individual of any age taking the test was 10 ± 3 (mean ± S.D.). Those whose score dropped by 1 S.D. (3 or more) from their pre-operative score were considered to be in the decline group. All other patients were assigned to the stable group.

The Mann Whitney U test was then used to assess differences in structural volumes between the stable and the decline groups for each neuropsychological test, as the normality distribution required for the use of t-test was not met. The Mann Whitney U test was also used to assess differences of the post-operative neuropsychological testing interval between stable and decline groups. Pearson’s correlation coefficient was used to explore the relationship between absolute score change and the post-operative neuropsychological testing interval.

Finally, linear regression modeling was used to examine the relationship of pre-surgical test scores to structural volumetric data and clinical variables. The pre-surgical test score of interest was used as the outcome variable. The structural volumes and clinical variables of interest as predictors. These predictors were examined individually due to the potential correlation between them.

## Results

The average (± S.D.) volume of the left and right thalamus was 0.59 ± 0.09 and 0.57 ± 0.09 (percentage of total intracranial volume), respectively. Additionally, the average (± S.D.) volume of the left and right hippocampus was 0.29 ± 0.05 and 0.28 ± 0.05 (percentage of total intracranial volume), respectively.

All three neuropsychology tests were associated with a statistically significant decline in scores following DBS; Immediate Story Recall (p<0.001), Delayed Story Recall (p = 0.001) and List Learning (p = 0.001). The results and mean score decline are included in Table 2.

**Table 2:** Neuropsychology test score decline.

Neuropsychology test	Mean Score Change	S.D.	Wilcoxon signed rank test (p value)
Delayed Story Recall (n = 38)*	-1.68	±2.74	0.01
Immediate Story Recall (n = 40)	-1.93	±2.72	<0.01
List Learning (n = 40)	-1.43	±2.19	0.02

Mean score change, standard deviation and the associated p-value, for each test, obtained from Wilcoxon signed rank test.

* Full Delayed Story Recall data was not available for two patients.

Age and duration of symptoms were selected as potential confounders of interest for the change in Delayed Story Recall score linear regression model, following prior testing with a univariate model (S1 Table). Similarly, duration of symptoms was selected as an additional confounder for the change in List Learning score model.

Unadjusted linear regression modeling did not demonstrate a relationship between structural volumetric data and verbal memory score change (Table 3). However, the Delayed Story Recall score change model showed important associations with the predictors (Left Thalamus, Right Thalamus, and Left Hippocampus), with considerably low p-values of, 0.06, 0.07, and 0.06 for the three predictors respectively.

**Table 3:** Linear modelling of change in test scores with volumetric and clinical data.

	Change in test score								
	*Delayed Story Recall*			*List Learning*			*Immediate Story Recall*		
Predictors	*β*	*Standard error*	*p-value*	*β*	*Standard error*	*p-value*	*β*	*Standard error*	*p-value*
*Left Thalamus*	9.37	4.80	0.06	5.57	3.76	0.15	4.97	4.72	0.30
	**Adjusted model**			**Adjusted model**					
*Left Thalamus*	7.12	4.90	0.16	8.84	3.74	**0.02**			
*Duration of Symptoms*	0.12	0.09	0.19	-0.16	0.67	**0.02**			
*Age*	-0.08	0.06	0.18						

*Right Thalamus*	9.62	5.22	0.07	4.98	4.08	0.23	4.36	5.11	0.40
	**Adjusted model**			**Adjusted model**					
*Right Thalamus*	6.86	5.39	0.21	4.16	8.36	0.05			
*Duration of Symptoms*	0.12	0.09	0.19	-0.16	0.07	**0.03**			
*Age*	-0.08	0.06	0.19						

*Left Hippocampus*	16.90	8.80	0.06	12.10	6.82	0.09	11.87	8.58	0.18
	**Adjusted model**			**Adjusted model**					
*Left Hippocampus*	12.58	8.92	0.17	16.13	6.72	**0.02**			
*Duration of Symptoms*	0.13	0.09	0.15	-0.15	0.07	**0.03**			
*Age*	-0.07	0.06	0.23						

*Right Hippocampus*	9.06	9.79	0.36	11.76	7.21	0.11	8.43	9.14	0.36
	**Adjusted model**			**Adjusted model**					
*Right Hippocampus*	4.25	9.76	0.67	15.91	7.12	**0.03**			
*Duration of Symptoms*	0.15	0.09	0.11	-0.15	0.07	**0.03**			
*Age*	-0.08	0.06	0.19						

Table to show the results of linear modelling investigating the relationship of test score change with volumetric and clinical data. The absolute test score change was used as the outcome variable. In the unadjusted model, the volume of the structure of interest was used as the predictor. In the adjusted model, the volume of the structure of interest and clinical variables, found to be of interest (see S1 Table) for the test in question were chosen as predictors. Significant p-values are highlighted in bold. *Duration of symptoms* was found to be a significant predictor of change in list learning score. *Left thalamic and hippocampal and right hippocampal volumes* were also found to be significant predictors of list learning score change in the adjusted models. Age was not a significant predictor of score change in any of the neuropsychological tests.

Adjustment was then made for age and/or duration of symptoms, as this was associated with the outcome, with a p value of <0.2, for the related verbal memory tests (List Learning and Delayed Story Recall), as demonstrated in Table 3. UPDRS Part III was not as associated with the outcome variables (p>0.2) in our initial testing (S1 Table). We did not therefore, adjust for this in subsequent analyses.

The *adjusted* List Learning score change regression model demonstrated that left thalamic (p = 0.02), left hippocampal (p = 0.02), right hippocampal (p = 0.03) volumes and duration of symptoms (p = 0.02 to 0.03) were significant predictors of post DBS score change (Table 3). Right thalamic volumetric data narrowly missed statistical significance (p = 0.05).

In our secondary analysis (Table 4), there was a significant difference of the left (p = 0.008) and right (p = 0.004) thalamic volumes between those in the decline group and those in the stable group for the Delayed Story Recall test. A significant difference was also noted with the hippocampal volumes (p = 0.01 for left and p = 0.04 for right hippocampus). There were no significant differences in volumes of structures between groups for the Immediate Story Recall or List Learning. Age was noted to be significantly different (p = 0.03) between decline and stable groups in the Immediate Story Recall analysis. Duration of symptoms was noted to significantly differ between groups for List Learning (p = 0.02).

**Table 4:** Volumetric differences between stable and decline performers.

Comparison of clinical and volumetric parameters between stable and decline performers in Delayed Story Recall (n = 38)^**♮**^			
**Parameters**	**Groups**		p-value
	Stable (n = 21)	Decline (n = 17)	
*Clinical*			
Age at surgery	61.1 ±7.4	58.2 ± 7.05	0.15
UPDRS Part III (n = 36)	36.0 ±15.4	40.1 ± 22.3	0.66
Duration of symptoms	12.6 ± 5.4	10.0 ± 4.3	0.20
Gender (M/F)	11/10	11/6	
*Volumetric (percent of TIV)*			
**Left Thalamus**	**0.62 ± 0.10**	**0.54 ± 0.06**	**0.008**
**Right Thalamus**	**0.60 ± 0.09**	**0.53 ± 0.05**	**0.004**
**Left Hippocampus**	**0.30 ± 0.05**	**0.27 ± 0.04**	**0.01**
**Right Hippocampus**	**0.29 ± 0.05**	**0.26 ± 0.04**	**0.04**
Comparison of clinical and volumetric parameters between stable and decline performers in Immediate Story Recall (n = 40)			
**Parameters**	**Groups**		p-value
	Stable (n = 22)	Decline (n = 18)	
*Clinical*			
**Age at surgery**	**57.9 ± 6.5**	**62.2 ±7.6**	**0.03**
UPDRS Part III *(n = 38)*	39.0 ± 16.6	39.3 ± 22.1	0.68
Duration of symptoms	12.7 ±5.4	12.2 (±5.6)	0.95
Gender (M/F)	12/10	11/7	
*Volumetric (percent of TIV)*			
Left Thalamus	0.60 ± 0.10	0.57 ± 0.08	0.27
Right Thalamus	0.58 ± 0.10	0.55 ± 0.07	0.26
Left Hippocampus	0.30 ± 0.05	0.27 ± 0.05	0.15
Right Hippocampus	0.30 ± 0.05	0.27 ± 0.05	0.18
Comparison of clinical and volumetric parameters between stable and decline performers in List Learning test (n = 40)			
**Parameters**	**Groups**		p-value
	Stable (n = 30)	Decline (n = 10)	
*Clinical*			
Age at surgery	59.7 ± 7.1	60.2 ± 8.1	0.77
UPDRS Part III *(n = 38)*	34.7 ± 15.0	51.7 ± 24.3	0.07
**Duration of symptoms**	**10.4 ± 4.4**	**15.2 ± 5.8**	**0.02**
Gender (M/F)	14/16	9/1	
*Volumetric (percent of TIV)*			
Left Thalamus	0.59 ± 0.08	0.57 ± 0.13	0.25
Right Thalamus	0.57 ± 0.07	0.56 ± 0.12	0.35
Left Hippocampus	0.29 ± 0.04	0.27 ± 0.07	0.10
Right Hippocampus	0.29 ± 0.05	0.27 ± 0.06	0.43

Table to show the clinical and volumetric parameters of stable and decline performers in the Story Recall Delayed, Immediate Story Recall and List Learning tests. A Mann-Whitney U Test was used to assess the difference between clinical and volumetric data between groups. Significant values are highlighted in bold.

^**♮**^2 patients were excluded as complete Story Recall Delayed test data was not available

No significant differences were seen between the duration of post-operative neuropsychological testing interval in stable and decline groups for all tests: Delayed Story Recall (p = 0.47), Immediate Story Recall (p = 0.72) and List Learning (p = 0.59). Furthermore, Pearson correlation coefficient did not show a significant correlation between post-operative neuropsychological testing interval and absolute score change for all tests: Delayed Story Recall (r = 0.07, p = 0.65), Immediate Story Recall (r = 0.17, p = 0.29) and List Learning (r = -0.12, p = 0.47).

Finally, linear regression modeling did not demonstrate a significant relationship between pre-surgical verbal memory scores and structural volumetric data nor with clinical variable data (S2 Table).

## Discussion

The results of our primary statistical analysis demonstrated that hippocampal (left, p = 0.02 and right, p = 0.03) and thalamic volumes (left p = 0.02) were significant predictors of List Learning score change following STN-DBS. Additionally, volumetric data of the left (p = 0.06) and right thalamus (p = 0.07) and left hippocampus (p = 0.06) suggested an important association with very low p-values, in Delayed Story Recall score change.

The results of our secondary analysis showed that following STN-DBS, patients in the decline group for the Delayed Story Recall test had significantly different left (p = 0.008) and right (p = 0.004) thalamic and left (p = 0.01) and right (p = 0.04) hippocampal volumes compared to those in the stable group.

During DBS-surgery, the electrode typically enters the brain via the prefrontal cortex and passes through the internal capsule and thalamus to reach the STN [21]. Furthermore, studies have suggested extensive reciprocal connections of the thalamus and the hippocampus [22–24]. Hence smaller volumes, and likely fewer reciprocal connections, may make these patients more susceptible to the effects of interruption or attenuation of these connections. This may be through direct effects, such as electrode trajectory or indirectly, through stimulation.

In one electrophysiology study [25], ventrolateral thalamus stimulation during the *presentation* of information lead to a decrease in recall error in a verbal memory task. However, in the same study, stimulation during the time of *recall*, increased recall error. The relationship of verbal memory to stimulation is not well delineated and may not be simply explained by the electrode trajectory.

Fronto-striatal dysfunction has been suggested as a factor in such deficits in List Learning scores in PD patients [26]. It is possible that such deficits are partially captured purely by the thalamic or hippocampal volumes. Further studies encompassing volumes of the striatal components may help elucidate their relationship to such deficits.

The hippocampus has been implicated in different memory functions including delayed verbal memory recall [27–28]. Hippocampal and thalamic volumes were however not found to be associated to Immediate Story Recall score changes. This test relies on information entering the patient’s cognition initially via the working memory. Thus it may be less sensitive to deficits in more long-term verbal memory, as tested by Delayed Story Recall. Additionally, the putamen has been suggested to play a role in the screening of information entering the working memory [29].

It may be possible that smaller pre-surgical structural volumes are related to lower pre-surgical score. In one MRI study of normal subjects [11] the volume of left thalamus linked to left temporal lobe was shown to correlate with verbal memory scores. However, in our analysis we did not find a significant relationship between pre-surgical volumes of the thalamus or hippocampus with pre-surgical scores. Hence, *whole* thalamic or hippocampal volumes may be a less sensitive indicator of verbal memory performance pre-operatively.

In addition to volumetric data, duration of symptoms was also noted to be a significant confounder related to List Learning score change (p = 0.02 to 0.03). Furthermore, in our secondary analysis, duration of symptoms was significantly different (p = 0.02) between stable and decline groups for List Learning. Age was found to be significantly different (p = 0.03) between stable and decline groups for Immediate Story Recall. STN-DBS may perhaps exacerbate the known relationship of disease progression and cognitive decline [30].

### Limitations

Firstly, the MR images used in this study were acquired from a 1.5T MRI scanner. Quality of images is an important limiting factor in the ability of the analysis software employed to reliably and accurately analyse brain MRI data. Thus 3T MRI may have produced better quality images for analysis. However, the analysed MRI images were acquired in frame and lacked head motion, which allowed good segmentation of sub-cortical structures to be achieved. Additionally, a bias field correction was also applied to the images.

Secondly, small image artefacts were present around the pin sites and their presence may have limited the accuracy of the SIENAX process. This can be overcome by using pre-op MRI without the frame in future.

Thirdly, we acknowledge that our sample size is small, lowering the power of the study. Furthermore, this is an observational study and therefore, no conclusions about causation are possible. In addition, we have conducted several sub-group analyses in this study. We argue nonetheless that our findings are new and important, they should however, be cautiously interpreted as hypothesis generating rather than confirmatory. Validation of these in larger samples will be required.

Fourthly, the follow-up period for the study was relatively short. The data used for the study was obtained from standard follow up visits following DBS surgery in our unit. There would be great interest in exploring relationships of volumetric data to neuropsychological outcomes over a longer follow up period.

Finally, the gold standard for hippocampal volume estimation is through manual segmentation. However, on visual inspection we believe good segmentation was achieved.

## Conclusion

In this paper we showed that following STN-DBS, verbal memory score change appeared to be associated with volumetric data of the thalamus and hippocampus and duration of symptoms. Additionally, patients with a decline in verbal memory score had smaller thalamic and hippocampal volumes compared to those whose scores remained stable. Duration of symptoms and age were also associated with verbal memory score change.

Pre-surgical verbal memory scores were not related to pre-surgical thalamic and hippocampus volumetric data nor to pre-operative clinical variables such as age, duration of symptoms or motor severity (UPDRS Part III score).

Whilst there is evidence for direct involvement of hippocampus and thalamus with the verbal memory, the true role of smaller volumes and their relationship to post-DBS score changes remain unclear. Further studies are needed to fully define the relationships of these structures to post-DBS verbal memory decline.

## Supporting Information

S1 Table Linear modelling of change in test score and clinical variables. Table to show the results of linear modelling investigating the relationship of test score change with age, duration of symptoms and UPDRS Part III score. The absolute test score change in the test of interest was used as the outcome variable. Age, duration of symptoms or UPDRS Part III score was used as the predictor. A p-value of <0.2 was used to determine those predictors which would be consequently used in the primary analysis for the related neuropsychological test (Table 3). Duration of symptoms was selected as an additional predictor in the consequent linear regression model for *change* in List Learning score. Age and duration of symptoms were selected as additional predictors in the consequent linear regression model for *change* in Delayed Story Recall score.
(DOCX)

S2 Table Linear modelling of pre-surgical test score and volumetric and clinical variables. Table to show linear modelling of pre-surgical test scores to structural volumetric data and clinical variables. The outcome variable was the pre-surgical test score of interest. The structural volume or clinical variable of interest was used as the predictor. No statistically significant relationships were demonstrated.
(DOCX)

## References

[1] Reetz K, Gaser C, Klein C, Hagenah J, Buchel C, Gottschalk S, et al Structural findings in the basal ganglia in genetically determined and idiopathic Parkinson's disease. Movement disorders: official journal of the Movement Disorder Society. 2009;24(1):99–103. 10.1002/mds.22333 . 18823048

[2] Nutt JG. Motor fluctuations and dyskinesia in Parkinson's disease. Parkinsonism & related disorders. 2001;8(2):101–8. . 11489675 10.1016/s1353-8020(01)00024-4

[3] Machado A, Rezai AR, Kopell BH, Gross RE, Sharan AD, Benabid AL. Deep brain stimulation for Parkinson's disease: surgical technique and perioperative management. Movement disorders: official journal of the Movement Disorder Society. 2006;21 Suppl 14:S247–58. 10.1002/mds.20959 . 16810722

[4] Anderson VC, Burchiel KJ, Hogarth P, Favre J, Hammerstad JP. Pallidal vs subthalamic nucleus deep brain stimulation in Parkinson disease. Archives of neurology. 2005;62(4):554–60. 10.1001/archneur.62.4.554 . 15824252

[5] Follett KA, Weaver FM, Stern M, Hur K, Harris CL, Luo P, et al Pallidal versus subthalamic deep-brain stimulation for Parkinson's disease. The New England journal of medicine. 2010;362(22):2077–91. 10.1056/NEJMoa0907083 . 20519680

[6] Saint-Cyr JA, Albanese A. STN DBS in PD: selection criteria for surgery should include cognitive and psychiatric factors. Neurology. 2006;66(12):1799–800. 10.1212/01.wnl.0000227468.17113.07 . 16801638

[7] Funkiewiez A, Ardouin C, Caputo E, Krack P, Fraix V, Klinger H, et al Long term effects of bilateral subthalamic nucleus stimulation on cognitive function, mood, and behaviour in Parkinson's disease. Journal of neurology, neurosurgery, and psychiatry. 2004;75(6):834–9. 15145995 10.1136/jnnp.2002.009803 PMC1739075

[8] Odekerken VJ, van Laar T, Staal MJ, Mosch A, Hoffmann CF, Nijssen PC, et al Subthalamic nucleus versus globus pallidus bilateral deep brain stimulation for advanced Parkinson's disease (NSTAPS study): a randomised controlled trial. Lancet neurology. 2013;12(1):37–44. 10.1016/S1474-4422(12)70264-8 . 23168021

[9] Yaguez L, Costello A, Moriarty J, Hulse N, Selway R, Clough C, et al Cognitive predictors of cognitive change following bilateral subthalamic nucleus deep brain stimulation in Parkinson's disease. Journal of clinical neuroscience: official journal of the Neurosurgical Society of Australasia. 2014;21(3):445–50. 10.1016/j.jocn.2013.06.005 . 24231557

[10] Beyer MK, Bronnick KS, Hwang KS, Bergsland N, Tysnes OB, Larsen JP, et al Verbal memory is associated with structural hippocampal changes in newly diagnosed Parkinson's disease. Journal of neurology, neurosurgery, and psychiatry. 2013;84(1):23–8. 10.1136/jnnp-2012-303054 23154124 PMC4041694

[11] Philp DJ, Korgaonkar MS, Grieve SM. Thalamic volume and thalamo-cortical white matter tracts correlate with motor and verbal memory performance. NeuroImage. 2014;91:77–83. 10.1016/j.neuroimage.2013.12.057 . 24401559

[12] Stewart CC, Griffith HR, Okonkwo OC, Martin RC, Knowlton RK, Richardson EJ, et al Contributions of volumetrics of the hippocampus and thalamus to verbal memory in temporal lobe epilepsy patients. Brain and cognition. 2009;69(1):65–72. 10.1016/j.bandc.2008.05.005 18599175 PMC2796537

[13] Hasegawa H, Samuel M, Douiri A, Ashkan K. Patients' Expectations in Subthalamic Nucleus Deep Brain Stimulation Surgery for Parkinson Disease. World neurosurgery. 2014 10.1016/j.wneu.2014.02.001 . 24518887

[14] Jenkinson M PM, Smith S. BET2: MR-based estimation of brain, skull and scalp surfaces. Eleventh Annual Meeting of the Organization for Human Brain Mapping; 2005; Toronto, Ontario, Canada.

[15] Smith SM, Zhang Y, Jenkinson M, Chen J, Matthews PM, Federico A, et al Accurate, robust, and automated longitudinal and cross-sectional brain change analysis. NeuroImage. 2002;17(1):479–89. . 12482100 10.1006/nimg.2002.1040

[16] Zhang Y, Brady M, Smith S. Segmentation of brain MR images through a hidden Markov random field model and the expectation-maximization algorithm. IEEE transactions on medical imaging. 2001;20(1):45–57. 10.1109/42.906424 . 11293691

[17] Patenaude B, Smith SM, Kennedy DN, Jenkinson M. A Bayesian model of shape and appearance for subcortical brain segmentation. NeuroImage. 2011;56(3):907–22. 10.1016/j.neuroimage.2011.02.046 21352927 PMC3417233

[18] Jenkinson M, Bannister P, Brady M, Smith S. Improved optimization for the robust and accurate linear registration and motion correction of brain images. NeuroImage. 2002;17(2):825–41. . 12377157 10.1016/s1053-8119(02)91132-8

[19] Smith SM, Jenkinson M, Woolrich MW, Beckmann CF, Behrens TE, Johansen-Berg H, et al Advances in functional and structural MR image analysis and implementation as FSL. NeuroImage. 2004;23 Suppl 1:S208–19. 10.1016/j.neuroimage.2004.07.051 . 15501092

[20] Costello A, Khamees HA, Moriarty J, Hulse N, Malik I, Selway R, et al Non-amnestic mild cognitive impairment is a prominent aspect in Parkinson’s disease patients being considered for deep brain stimulation. Basal Ganglia. 2011;1(4):213–20. 10.1016/j.baga.2011.10.003.

[21] Witt K, Daniels C, Volkmann J. Factors associated with neuropsychiatric side effects after STN-DBS in Parkinson's disease. Parkinsonism & related disorders. 2012;18 Suppl 1:S168–70. 10.1016/S1353-8020(11)70052-9 . 22166423

[22] Herkenham M. The connections of the nucleus reuniens thalami: evidence for a direct thalamo-hippocampal pathway in the rat. The Journal of comparative neurology. 1978;177(4):589–610. 10.1002/cne.901770405 . 624792

[23] Su HS, Bentivoglio M. Thalamic midline cell populations projecting to the nucleus accumbens, amygdala, and hippocampus in the rat. The Journal of comparative neurology. 1990;297(4):582–93. 10.1002/cne.902970410 . 1696591

[24] Wouterlood FG, Saldana E, Witter MP. Projection from the nucleus reuniens thalami to the hippocampal region: light and electron microscopic tracing study in the rat with the anterograde tracer Phaseolus vulgaris-leucoagglutinin. The Journal of comparative neurology. 1990;296(2):179–203. 10.1002/cne.902960202 . 2358531

[25] Ojemann GA, Blick KI, Ward AA Jr. Improvement and disturbance of short-term verbal memory with human ventrolateral thalamic stimulation. Brain: a journal of neurology. 1971;94(2):225–40. . 4936864 10.1093/brain/94.2.225

[26] Zahodne LB, Bowers D, Price CC, Bauer RM, Nisenzon A, Foote KD, et al The case for testing memory with both stories and word lists prior to dbs surgery for Parkinson's Disease. The Clinical neuropsychologist. 2011;25(3):348–58. 10.1080/13854046.2011.562869 21491347 PMC3077807

[27] Reitz C, Brickman AM, Brown TR, Manly J, DeCarli C, Small SA, et al Linking hippocampal structure and function to memory performance in an aging population. Archives of neurology. 2009;66(11):1385–92. 10.1001/archneurol.2009.214 19901171 PMC2778802

[28] Schmidt-Wilcke T, Poljansky S, Hierlmeier S, Hausner J, Ibach B. Memory performance correlates with gray matter density in the ento-/perirhinal cortex and posterior hippocampus in patients with mild cognitive impairment and healthy controls—a voxel based morphometry study. NeuroImage. 2009;47(4):1914–20. 10.1016/j.neuroimage.2009.04.092 . 19442751

[29] Baier B, Karnath HO, Dieterich M, Birklein F, Heinze C, Muller NG. Keeping memory clear and stable—the contribution of human basal ganglia and prefrontal cortex to working memory. The Journal of neuroscience: the official journal of the Society for Neuroscience. 2010;30(29):9788–92. 10.1523/JNEUROSCI.1513-10.2010 . 20660261 PMC6632833

[30] Pigott K, Rick J, Xie SX, Hurtig H, Chen-Plotkin A, Duda JE, et al Longitudinal study of normal cognition in Parkinson disease. Neurology. 2015;85(15):1276–82. 10.1212/WNL.0000000000002001 26362285 PMC4617168

