# Supplementary material for: Verbal Memory Decline following DBS for Parkinson’s Disease: Structural Volumetric MRI Relationships
Source: PLoS One. 2016 Aug 24;11(8):e0160583. doi: 10.1371/journal.pone.0160583 (PMC4996448; doi:10.1371/journal.pone.0160583)
Supplement: S1 Table — Table to show the results of linear modelling investigating the relationship of test score change with age, duration of symptoms and UPDRS Part III score. The absolute test score change in the test of interest was used as the outcome variable. Age, duration of symptoms or UPDRS Part III score was used as the predictor. A p-value of <0.2 was used to determine those predictors which would be consequently used in the primary analysis for the related neuropsychological test (Table 3). Duration of symptoms was selected as an additional predictor in the consequent linear regression model for change in List Learning score. Age and duration of symptoms were selected as additional predictors in the consequent linear regression model for change in Delayed Story Recall score. (DOCX) [file pone.0160583.s001.docx]

**S1 Table. Linear modelling of change in test score and clinical variables.**

| **Score change** | **Age** | | |
| --- | --- | --- | --- |
|  | *β* | *Standard error* | *p-value* |
| *Immediate Story Recall* | -0.04 | 0.06 | 0.51 |
| ***Delayed Story Recall*** | **-0.09** | **0.06** | **0.18** |
| *List Learning* | 0.05 | 0.05 | 0.32 |
| **Score change** | **Duration of symptoms** | | |
|  | *β* | *Standard error* | *p-value* |
| *Immediate Story Recall* | 0.06 | 0.09 | 0.45 |
| ***Delayed Story Recall*** | 0.15 | 0.09 | **0.09** |
| ***List Learning*** | -0.11 | 0.07 | **0.12** |
| **Score change** | **UPDRS Part III score** | | |
|  | *β* | *Standard error* | *p-value* |
| *Immediate Story Recall* | -0.01 | 0.02 | 0.75 |
| *Delayed Story Recall* | -0.01 | 0.03 | 0.80 |
| *List Learning* | -0.20 | 0.02 | 0.30 |

Table to show the results of linear modelling investigating the relationship of test score change with age, duration of symptoms and UPDRS Part III score. The absolute test score change in the test of interest was used as the outcome variable. Age, duration of symptoms or UPDRS Part III score was used as the predictor. A p-value of <0.2 was used to determine those predictors which would be consequently used in the primary analysis for the related neuropsychological test (Table 3). Duration of symptoms was selected as an additional predictor in the consequent linear regression model for *change* in List Learning score. Age and duration of symptoms were selected as additional predictors in the consequent linear regression model for *change* in Delayed Story Recall score.
