# Supplementary material for: Verbal Memory Decline following DBS for Parkinson’s Disease: Structural Volumetric MRI Relationships
Source: PLoS One. 2016 Aug 24;11(8):e0160583. doi: 10.1371/journal.pone.0160583 (PMC4996448; doi:10.1371/journal.pone.0160583)
Supplement: S2 Table — Table to show linear modelling of pre-surgical test scores to structural volumetric data and clinical variables. The outcome variable was the pre-surgical test score of interest. The structural volume or clinical variable of interest was used as the predictor. No statistically significant relationships were demonstrated. (DOCX) [file pone.0160583.s002.docx]

**S2 Table. Linear modelling of pre-surgical test score and volumetric and clinical variables.**

| **Predictors** | **Pre-surgical Delayed Story Recall score** | | |
| --- | --- | --- | --- |
|  | *β* | *Standard error* | *p-value* |
| *Left Thalamus* | -4.25 | 5.17 | 0.42 |
| *Right Thalamus* | -5.10 | 5.56 | 0.37 |
| *Left Hippocampus* | -0.80 | 9.57 | 0.93 |
| *Right Hippocampus* | 0.24 | 10.1 | 0.98 |
| *Age at surgery* | 0.05 | 0.07 | 0.46 |
| *UPDRS Part III score* | -0.02 | 0.03 | 0.37 |
| *Duration of symptoms* | -0.14 | 0.09 | 0.13 |
| **Predictors** | **Pre-surgical List Learning score** | | |
|  | *β* | *Standard error* | *p-value* |
| *Left Thalamus* | -1.93 | 4.91 | 0.70 |
| *Right Thalamus* | -2.61 | 5.28 | 0.62 |
| *Left Hippocampus* | 2.83 | 9.02 | 0.76 |
| *Right Hippocampus* | 3.15 | 9.48 | 0.74 |
| *Age at surgery* | -0.10 | 0.06 | 0.13 |
| *UPDRS Part III score* | -0.04 | 0.02 | 0.15 |
| *Duration of symptoms* | 0.04 | 0.09 | 0.66 |
| **Predictors** | **Pre-surgical Immediate Story Recall score** | | |
|  | *β* | *Standard error* | *p-value* |
| *Left Thalamus* | -2.67 | 5.38 | 0.62 |
| *Right Thalamus* | -2.95 | 5.79 | 0.61 |
| *Left Hippocampus* | -1.10 | 9.91 | 0.91 |
| *Right Hippocampus* | -2.09 | 10.4 | 0.84 |
| *Age at surgery* | -0.03 | 0.07 | 0.96 |
| *UPDRS Part III score* | -0.04 | 0.03 | 0.16 |
| *Duration of symptoms* | -0.10 | 0.10 | 0.28 |

Table to show linear modelling of pre-surgical test scores to structural volumetric data and clinical variables. The outcome variable was the pre-surgical test score of interest. The structural volume or clinical variable of interest was used as the predictor. No statistically significant relationships were demonstrated.
